# Assessment of Viral Targeted Sequence Capture Using Nanopore Sequencing Directly from Clinical Samples

**DOI:** 10.3390/v12121358

**Published:** 2020-11-27

**Authors:** Leonard Schuele, Hayley Cassidy, Erley Lizarazo, Katrin Strutzberg-Minder, Sabine Schuetze, Sandra Loebert, Claudia Lambrecht, Juergen Harlizius, Alex W. Friedrich, Silke Peter, Hubert G. M. Niesters, John W. A. Rossen, Natacha Couto

**Affiliations:** 1 Department of Medical Microbiology and Infection Prevention, University Medical Center Groningen, University of Groningen, 9713 RC Groningen, The Netherlands; h.j.cassidy@umcg.nl (H.C.); e.f.lizarazo.forero@umcg.nl (E.L.); alex.friedrich@umcg.nl (A.W.F.); h.g.m.niesters@umcg.nl (H.G.M.N.); john.rossen@gmail.com (J.W.A.R.); nmgdc20@bath.ac.uk (N.C.); 2 Institute of Medical Microbiology and Hygiene, University of Tübingen, 72076 Tübingen, Germany; Silke.Peter@med.uni-tuebingen.de; 3 IVD Innovative Veterinary Diagnostics (IVD GmbH), 30926 Seelze, Germany; Strutzberg@ivd-gmbh.de; 4 Animal Health Services, Chamber of Agriculture of North Rhine-Westphalia, 59505 Bad Sassendorf, Germany; Sabine.Schuetze@LWK.NRW.DE (S.S.); Sandra.Loebert@lwk.nrw.de (S.L.); Claudia.Lambrecht@LWK.NRW.DE (C.L.); Juergen.Harlizius@LWK.NRW.DE (J.H.); 5 Department of Pathology, University of Utah School of Medicine, Salt Lake City, UT 84108, USA; 6 Milner Centre for Evolution, Department of Biology and Biochemistry, University of Bath, Bath BA2 7AY, UK

**Keywords:** next-generation sequencing, one health, shotgun metagenomic sequencing, porcine viruses, targeted sequence capture, viral metagenomics, virome

## Abstract

Shotgun metagenomic sequencing (SMg) enables the simultaneous detection and characterization of viruses in human, animal and environmental samples. However, lack of sensitivity still poses a challenge and may lead to poor detection and data acquisition for detailed analysis. To improve sensitivity, we assessed a broad scope targeted sequence capture (TSC) panel (ViroCap) in both human and animal samples. Moreover, we adjusted TSC for the Oxford Nanopore MinION and compared the performance to an SMg approach. TSC on the Illumina NextSeq served as the gold standard. Overall, TSC increased the viral read count significantly in challenging human samples, with the highest genome coverage achieved using the TSC on the MinION. TSC also improved the genome coverage and sequencing depth in clinically relevant viruses in the animal samples, such as influenza A virus. However, SMg was shown to be adequate for characterizing a highly diverse animal virome. TSC on the MinION was comparable to the NextSeq and can provide a valuable alternative, offering longer reads, portability and lower initial cost. Developing new viral enrichment approaches to detect and characterize significant human and animal viruses is essential for the One Health Initiative.

## 1. Introduction

Genomic identification and characterization of viruses in both humans and animals play a central role in the diagnosis, monitoring and control of infectious diseases [1]. Clinical diagnostics remains a challenge in infections with indiscriminate clinical presentation, coupled with pathogen diversity and microbial abundance in clinical samples [2]. New approaches to identify potentially significant viruses in humans and animals (e.g., pigs, important mixing vessels) are a crucial part of the One Health Initiative. With the increasing global population and intensification of agriculture, zoonotic pathogen spillover at the human–animal–wildlife interface, such as the recent outbreaks of SARS-CoV-2 [3] and swine influenza virus (SIV) [4], remains a challenge.

Metagenomic testing allows for the detection of pathogens directly from clinical specimens without *a priori* knowledge of target sequences. Metagenomics uses genome sequencing and bioinformatics techniques to identify and characterize microorganisms, including viruses, fungi and bacteria [5,6]. The approach makes it a promising tool after targeted conventional methods fail [7]. On the other hand, the untargeted nature of SMg entails the sequencing of non-pathogenic, host and environmental nucleic acids, along with sequences of interest. This can result in an overall reduced sensitivity compared to conventional targeted approaches, such as qPCR [6].

Pre-lysis enrichment procedures such as centrifugation and/or filtration have been reported to increase viral sensitivity [8]. However, pre-lysis procedures rely on the structural integrity of the microbial and host background cells. Due to the high costs of SMg, it is often applied retrospectively on selected and usually clinically relevant frozen samples after other diagnostic tests have failed. Alternatively, post-lysis enrichment procedures including DNase treatment [9], targeted PCR amplicon sequencing [7] and viral targeted sequence capture (TSC) offer depletion of background nucleic acids and improve the recovery of viral reads. Targeted capture probes are more tolerant of mismatches in target sequences than PCR primers, making them suitable for use with highly diverse targets such as RNA viruses, and have been reported to increase the number of viral reads and maintain viral diversity [10]. For example, the ViroCap share developer TSC panel from KAPA (Roche) contains approximately 2 million capture probes from all vertebrate viral genomes known in 2014 [11]. These oligonucleotide bait probes hybridize with target viral nucleic acids and isolate them from background nucleic acids using magnetic streptavidin-coated beads [7].

Currently, developed TSC panels such as those from KAPA (ViroCap) [11,12], Illumina and TwistBio are available for next-generation sequencing (NGS) Illumina platforms. Although highly adapted in clinical microbiology [13], Illumina sequencing is limited by short sequencing reads (≤2× 300 bp) [14]. Oxford Nanopore Technologies (ONT) sequencing platforms offer comparatively lower accuracy and output but provide long reads that can be analyzed in real-time. Moreover, with the low initial cost of the MinION sequencing device, ONT has steadily edged its way to the broader scientific community [15]. However, to the best of our knowledge, the only (commercial) sequence capture assay tested with ONT platforms is the Agilent SureSelect (Sequence capture (SQK-LSK109) Version: SCE_9075_v109_revO_14Aug2019) on human DNA cancer panels (https://nanoporetech.com/resource-centre/incorporating-sequence-capture-library-preparation-minion-gridion-and-promethion-0) and custom TSC probes on spiked viruses in cell-culture samples [16,17].

In this study, the ViroCap TSC panel was evaluated for the detection and characterization of viruses in challenging human and animal samples using long-read sequencing (LRS) on the ONT MinION device—firstly, by comparing it to a shotgun metagenomic approach on the MinION and secondly, by comparing it with the established ViroCap workflow on the Illumina NextSeq as a gold standard.

## 2. Materials and Methods

### 2.1. Sample Collection

Human and animal samples were collected to evaluate the applicability of the ViroCap TSC panel which covers a broad range of viruses. Four samples from transplant patients, presenting either respiratory or gastrointestinal symptoms, were selected based on a positive qPCR result [18] for two clinically relevant viruses: enterovirus (*n* = 4) and norovirus (*n* = 1) (Table 1). Conventional routine Sanger sequencing was able to characterize norovirus as GII.4; however, genotypes could not be determined for the enteroviruses. Four pig samples (each consisting of 5 pooled pig samples) were collected from farms within the German/Dutch border region between 2017 and 2018 for the Food Pro-tec-ts project. Samples were selected based on clinically relevant qPCR results from both non-symptomatic (*n* = 2) and symptomatic pigs (*n* = 2) (Table 1). qPCR was performed to screen for SIV (VetMAX™-Gold SIV Detection Kit) (Life Technologies, Carlsbad, CA, USA) and porcine reproductive and respiratory syndrome virus (PRRSV) (Virotype^®^ PRRSV RT-PCR Kit) (Qiagen, Hilden, Germany), according to the manufacturer’s recommendations.

### 2.2. Nucleic Acid Extraction and cDNA Synthesis

Human samples were initially centrifuged at 6000× *g* for 2 min. A total of 190 µL of the supernatant was isolated using the easyMAG (bioMérieux, Inc., Marcy l’Etoile, France) and eluted in 110 µL of elution buffer. Pig samples were centrifuged at 6000× *g* for 2 min. A total of 240 µL of supernatant was isolated using the QIAamp Viral RNA Mini Kit (Qiagen), with carrier RNA replaced by linear polyacrylamide and eluted in 90 µL of elution buffer. Lysis buffer was used as a negative control. Nucleic acids were cleaned and concentrated to 8 µL using the RNA clean and concentrator kit (Zymo Research, Irvine, CA, USA), including an in-column DNase treatment, according to the manufacturer’s recommendations.

**Figure 1:**
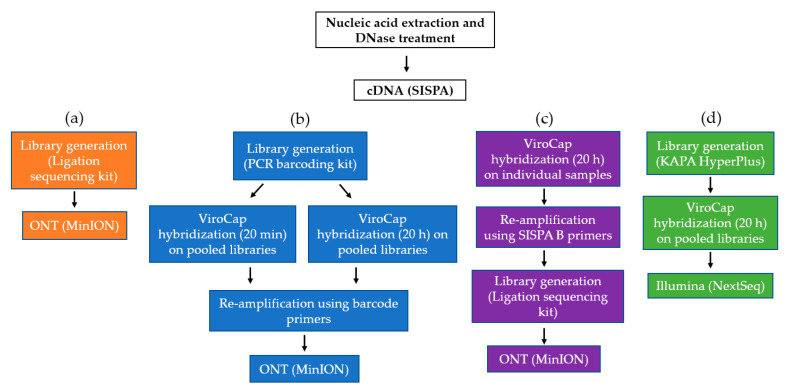
Workflow of the laboratory procedures evaluated in this study. (**a**) Shotgun approach for a baseline on Oxford Nanopore Technologies (ONT) platforms; (**b**) ViroCap on pooled barcoded library pools using a 20 min and 20 h hybridization time; (**c**) ViroCap on individual samples using a 20 h hybridization time; (**d**) standard ViroCap protocol on Illumina platforms. Abbreviations: ONT, Oxford Nanopore Technologies; SISPA, Sequence-Independent Single-Primer-Amplification.

Sequence-Independent Single-Primer-Amplification (SISPA) offers amplification and recovery of low biomass samples [19], while offering great flexibility. It can be utilized for both Illumina and ONT platforms [14]. Reverse transcription and generation of the second strand cDNA were performed as described [8]. Amplification of cDNA was performed as previously described [14]. Briefly, 5 μL of cDNA was amplified with AccuTaq LA (Sigma, Poole, United Kingdom) and 1 μL (100 pmol/μL) Sol-Primer B (5′-GTTTCCCACTGGAGGATA-3′) in a total reaction volume of 50 μL, according to the manufacturer’s recommendation. PCR reaction conditions were as follows: 98 °C for 30 s; 30 cycles of 94 °C for 15 s, 50 °C for 20 s and 68 °C for 5 min, followed by 68 °C for 10 min. The SISPA cDNA was then used as an untargeted basis for all three approaches; shotgun metagenomic sequencing on the MinION (M) (Figure 1a), viral TSC with ViroCap on the MinION (MV) (Figure 1b,c) and viral TSC with ViroCap on the NextSeq (NV) (Figure 1d).

### 2.3. Oxford Nanopore Technologies SMg and TSC

To evaluate TSC for ONT platforms, the untargeted SISPA cDNA served as a baseline (Figure 1a). Sequencing libraries were generated with the Ligation Sequencing Kit (SQK-LSK109) (ONT) and native barcoding expansion (EXP-NBD104) (ONT) using a modified One-pot protocol [20] (for details see: dx.doi.org/10.17504/protocols.io.bbnmimc6).

To create enriched long-read sequences using ONT, the ViroCap share developer panel (SeqCap EZ HyperCap Workflow User’s Guide version 2.1) from Roche NimbleGen (Madison, WI, USA) was used [11]. Viral TSC was evaluated on pooled samples (*n* = 8) (standard protocol) and individual samples (*n* = 8) (Figure 1b,c). For the pooled approach (Figure 1b), sequencing libraries were first generated from 8 samples using the PCR barcoding kit (SQK-PBK004) (ONT, Oxford, England) and pooled together prior to TSC. The captured library pool was then re-amplified using 0.4 µL of each of the respective barcode primers and sequenced using the MinION device. To reduce the overall turn-over time, the impact of a shorter hybridization time of 20 min was also evaluated to the recommended 20 h using the pooled cDNA (Figure 1b).

For the individual approach (Figure 1c), the following changes were applied to the ViroCap TSC protocol to adjust for long-read cDNA. The user guide was followed starting from the hybridization procedure (chapter 5) using the SeqCap EZ Developer Reagent, while the blocking oligos (for the KAPA library prep adapters) were not added. Next, the initial denaturation time for the hybridization incubation was reduced from 5 min to 45 sec (step 22). Washing and recovery steps were followed (chapter 6), ending with eluting the captured cDNA off the beads with 20 µL of water (step 29) prior to re-amplification. Captured cDNA was re-amplified using the round B SISPA primer and amplification parameters, as described previously [14]. This was followed by sequence library generation with the Ligation Sequencing Kit (SQK-LSK109) (ONT) and native barcoding expansion (EXP-NBD104) (ONT) using a modified One-pot protocol [20]. Moreover, a 50% dilution of recommended reagents and sample input quantities (to keep the same probe/sample ratio) were applied for individual sample TSC reactions to reduce the cost per sample. The eight individual TSC libraries were pooled together by equal mass prior to sequencing on the MinION device. For each approach (TSC on pooled or individual samples), the same eight samples (Table 1) and a negative control were sequenced on a MinION device (ONT) on FLO-MIN106 R9.4.1 flow cells (ONT).

### 2.4. Illumina TSC

To create enriched short-read sequencing (SRS) libraries for Illumina, the ViroCap share developer panel from Roche NimbleGen (Madison, WI, USA) was also used (Figure 1d). The SeqCap EZ HyperCap Workflow User’s Guide version 2.1 was followed according to the manufacturer’s recommendation. Briefly, eight sequence libraries and a negative control library were pooled prior to a target sequence capture reaction. A probe hybridization time of 20 h was selected. The captured library pool was then sequenced on an Illumina NextSeq 500 (Illumina, San Diego, CA, USA) with a medium output kit to generate paired-end 76 bp reads.

### 2.5. Data Analysis

ONT reads were base-called with Guppy v3.2.10 and trimmed using Porechop v0.2.4 (https://github.com/rrwick/Porechop). Illumina reads were trimmed using CLC Genomics Server 20.0.3 (Qiagen) (referred to as CLC from here on) using the default settings, with the quality limit set to 0.05. SISPA primer sequences were then removed from ONT and Illumina reads using the Trim adapter list on CLC. Library quality control (QC) metrics were derived from BaseSpace for SRS and from FastQC for LRS. For read-based taxonomic analysis, trimmed reads were uploaded using the web-based tool Taxonomer [21] and analyzed in full analysis mode. Additionally, the reads were mapped against an in-house viral database consisting of 67,324 complete viral sequences derived from GenBank on the 13/08/2019 using CLC with 80% nucleotide identity and 70% length fraction. The resulting consensus sequences were subsequently confirmed by NCBI BLASTn. To account for possible barcode cross-contamination and crosstalk, a cut-off was applied based on 0.1% of the total reads for that virus [22]. Coverage and read tracks were derived using CLC, by mapping the reads against the best viral hit using 80% nucleotide identity and 70% length fraction. Antiviral susceptibility to influenza virus was investigated using the online tool Influenza Research Database (https://www.fludb.org).

### 2.6. Ethics Statement

The human samples used for this study were collected during routine diagnostics and infection prevention control. Oral consent for the use of such clinical samples for research purposes was routinely obtained upon patient admission to the UMCG, in accordance with the guidelines of the Medical Ethics Committee of the University Medical Center Groningen. All experiments were performed in accordance with the guidelines of the Declaration of Helsinki and the institutional regulations, and all samples were anonymized. The animal samples used for this study were collected within the Food Pro-tec-ts project, which has been classified as an animal study and was approved on 22.09.2017 by the respective state office for nature, environment and consumer protection (file reference: 84.02.05.40.17.079).

### 2.7. Data Availability

Sequencing reads from all the approaches have been deposited at Sequence Read Archive under the BioProject number: PRJNA670157.

## 3. Results

### 3.1. Hybridization Time and Sample Pooling on the MinION

The increased sample handling and hybridization time are important limitations of TSC. To evaluate the impact of probe hybridization time on the MinION, samples were pooled before a 20 min and 20 h hybridization time. Pooling samples prior to a 20 h hybridization time is recommended in the ViroCap User’s Guide v.2.1. TSC was also performed on individual samples (using a hybridization time of 20 h), as a result of unspecific bindingobserved after amplification of captured library pools. The individual sample fastq files were concatenated to compare the number of viral reads with the 20 min and 20 h pools (Table 2). Sequence reads were mapped against an in-house viral database on CLC and normalized.

Interestingly, a hybridization time of 20 min resulted in the highest percentage of viral reads with 87.66%, while 20 h resulted in 76.53%. TSC reactions of individual samples (that were loaded onto the MinION by equal mass) resulted in the lowest percentage of viral reads (53.60%). Differences in viral loads could account for the lower percentage of viral reads in the individual TSC reactions. Viruses with high loads (e.g., influenza A virus (IAV) with a Ct of 19) could result in an overrepresentation bias in the pooled samples. Indeed, reads from IAV represented more than half of the total viral reads in both pooled reactions (20 min and 20 h). Meanwhile, in the individual capture reactions (in which libraries were loaded by equal mass), IAV reads accounted for 9.39% of viral reads. Therefore, the individual reaction had a more even IAV read representation.

Some viruses benefited from the shorter hybridization time of 20 min, such as ungulate tetraparvovirus 3 (10.02% of viral reads after 20 min, 0.44% after 20 h and 5.24% after 20 h individual). For other viruses, a shorter hybridization time seemed to have a negative impact, particularly in the human samples. Considerable differences were detected between the individual and pooled approaches in the human samples, with norovirus GII.4, enterovirus D68 (EV-D68), enterovirus A71 (EV-A71) and coxsackievirus A22 (CV-A22) yielding a higher percentage of viral reads from the individual approach. This study therefore highlights important differences to be considered when investigating different hosts and sample origins.

Significantly, we also observed that post-capture re-amplification of library pools using the ONT PCR barcoding kit proved to be too unspecific, resulting in excessive barcode crosstalk. As a result, an individual sample reaction with 20 h hybridization time was selected as the best option to proceed.

### 3.2. TSC and Taxonomic Binning

**Figure 2:**
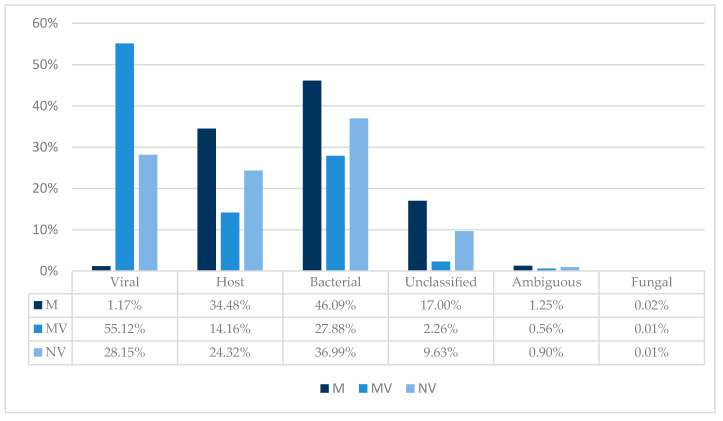
Sample composition with and without TSC on the ONT MinION and with TSC on the Illumina NextSeq. Abbreviations: M, MinION; MV, MinION with ViroCap; NV, NextSeq with ViroCap.

To illustrate the impact of TSC on a microbial and host nucleic acid background, trimmed reads were uploaded to Taxonomer to obtain sample compositions. The following graph provides a comparison overview between the approaches; ONT MinION device (M), ONT MinION device with ViroCap (MV) and Illumina NextSeq with ViroCap (NV) (Figure 2).

Shotgun metagenomic cDNA sequenced on the MinION (M) resulted in 1.17% viral reads, while MV increased the percentage of viral reads to 55.12%. Interestingly, NextSeq TSC resulted in a viral read count of 28.15%. This could suggest that longer unfragmented cDNA might be captured more efficiently. Prior to TSC, background host nucleic acid accounted for 34.48% of reads using the MinION. This decreased to 14.16% using TSC on the MinION. Meanwhile, background host nucleic acids accounted for 24.32% using TSC on the NextSeq. Furthermore, while bacterial nucleic acids accounted for 46.09% of MinION sequencing reads, they accounted for only 36.99% of reads after using TSC. Additionally, MV reduced the number of unclassified reads, compared to NV. Finally, an increased Q score was found using captured libraries compared to metagenomic libraries on the MinION (Table S1).

### 3.3. Viral Genome Coverage and Sequencing Depth

TSC can not only be used to increase the sensitivity of virus detection, but also to increase the genome coverage and sequencing depth. Sensitivity is particularly important in challenging patient samples with a low viral load, or samples with a high viral diversity such as pig samples. To assess genome coverage and genome coverage depth (sequencing depth) between the three approaches, trimmed reads were mapped against representative genome references using CLC (Table 3). As the MinION device produces a lower output compared to the NextSeq, the data were not normalized to reflect a realistic outcome of sequencing runs using both approaches.

Overall, 27 viruses were detected; four in the human samples and 23 in the animal samples (Table 3). Despite a lower read count generated using the ONT platform (Table S1), only porcine bocavirus 5 was not detected, compared to the Illumina NextSeq. Moreover, viruses that were not part of the ViroCap panel were still detected, albeit with fewer reads in some viruses (e.g., astrovirus wild boar/WBAstV-1 and atypical porcine pestivirus 1) following targeted capture. While the average genome coverage was lower in the MV animal libraries (63%) compared to the M libraries (67%), the sequencing depth of MV was considerably higher, indicating coverage bias (Table 3). Yet, MV yielded more clinically relevant near full-length genomes. Overall, ViroCap increased the average sequencing depth of 4/4 detected viruses in the human samples and 13/22 detected viruses in the animal samples on the MinION. Illumina sequencing (NV), compared to MinION with ViroCap, yielded the highest average sequencing depth.

### 3.4. Viral Detection in Human Samples

Four viruses were detected with each approach. Although the patient viruses were detected in routine diagnostics, the enteroviruses were recorded as untypeable. Both the shotgun metagenomic and TSC approaches enabled the characterization of enteroviruses in greater detail, highlighting a well-established advantage of NGS [7]. ViroCap resulted in a substantial increase in the number of viral reads and sequencing depth in all human samples. Three full-length genomes were recovered by MV (EV-D68, norovirus GII.4 and CV-A22), while only one full-length genome (norovirus GII.4) and one near full-length genome (EV-D68) were recovered by NV. The shotgun metagenomic approach on the MinION (M) did not perform as well, with only one near full-length genome recovered (norovirus GII.4). None of the approaches were able to detect the enterovirus from the Ct 30 human fecal sample (H2), suggesting a limit of detection. Enterovirus A71 (Ct 29) was detected (albeit at lower coverage) with each approach, supporting a potential cut-off in fecal samples. A co-infection was detected in human sample 3; norovirus GII.4 and enterovirus A71. It is interesting to note that while a shotgun metagenomic approach covered 20% of the EV-A71 genome, this was in fact accounted for by only one single long read. TSC increased the genome coverage and sequencing depth on both ONT and Illumina platforms and could confirm the presence of EV-A71.

### 3.5. Viral Detection in Animal Samples

The animal samples, on the other hand, proved to be highly diverse. We detected 22 viruses using both ONT approaches, while we detected 23 viruses using Illumina. Indeed, porcine bocavirus 5 was only detected by TSC on the NextSeq. M yielded four full-length genomes, while targeted capture yielded six and four using the MV and NV, respectively. Regarding near full-length genomes, M yielded six, MV yielded one and NV yielded four. Furthermore, MV increased the genome coverage in 9/22 viruses, compared to M. All approaches enabled us to determine *in silico* susceptibility of IAV (H1N1) to the neuraminidase inhibitors—Oseltamivir, Zanamivir and Peramivir—with 100% concordance.

### 3.6. ONT Accuracy

To assess the accuracy of ONT sequencing, selected viral species with a high sequencing depth (≥1457) were compared to the corresponding Illumina consensus sequences, which served as a gold standard (Table 4). ONT achieved an consensus accuracy of 98.71–99.89% (average 99.44%) compared to the NextSeq ViroCap consensus sequences.

### 3.7. Coverage Depth of Clinically Relevant Viruses

**Figure 3:**
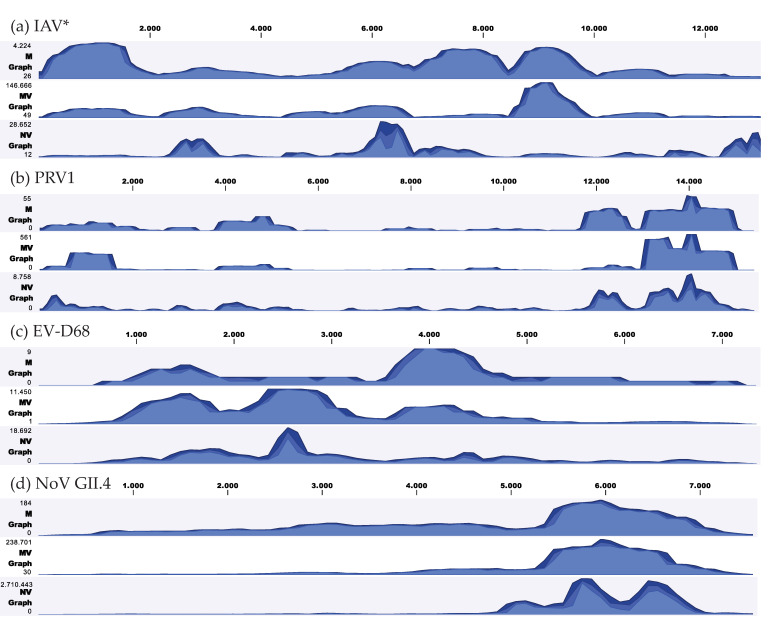
Visualization of the coverage depth for four clinically relevant viruses detected in our study. (**a**) Influenza A virus; (**b**) Porcine respirovirus 1; (**c**) Enterovirus D68; (**d**) Norovirus GII.4. Reads were not normalized and therefore actual depth was dependent on the number of total reads of the sample. * All 8 influenza A virus segments were concatenated into a single genome. Abbreviations: M, MinION; MV, MinION with ViroCap; NV, NextSeq with ViroCap; IAV, influenza A virus; PRV1, porcine respirovirus 1; EV-D68, enterovirus D68; NoV GII.4, norovirus GII.4.

To visualize and compare coverage patterns and sequencing depths between M, MV and NV, reads were mapped across clinically relevant viral genomes detected in our study. Figure 3 indicates differences between human and animal samples in terms of genome coverage, following TSC.

Apart from IAV, in which the NextSeq showed a different pattern, most other viruses in Figure 3 display a similar pattern before and after enrichment. This indicates that the coverage pattern is somewhat more dependent on the SISPA approach than on enrichment or the sequencing platform. Interestingly, after using TSC, a high sequencing depth was obtained for the previously untypeable Sanger sequencing enterovirus target, VP1 (position 2300–3300 bp).

## 4. Discussion

SMg has the potential for broad-range detection and characterization of viruses but is hindered by sample complexity, which can reduce viral sensitivity. Therefore, applying TSC such as ViroCap has been shown to increase virus detection [11,12,22]. While current Illumina-based NGS platforms offer high accuracy and sequencing depth, factors such as speed, mobility, sequence length and flexibility are disadvantageous compared to the ONT platforms.

Here, we show that a viral target enrichment assay designed for second generation SRS platforms can also be used with LRS ONT platforms, with only slight modifications. To assess the performance of TSC on ONT platforms, we also ran Illumina deep sequencing as a gold standard on challenging clinical samples. Moreover, SMg without enrichment was also performed using ONT. The SISPA methodology was selected as it offers robustness and flexibility and can be used both for Illumina and ONT platforms [14]. Due to the broad scope of ViroCap, it has also been reported to enrich uncommon viruses [11]. Therefore, we also evaluated the benefits and drawbacks of TSC in highly diverse animal samples next to human samples. While a TSC panel was evaluated in farm animals on the Illumina platform previously [23], to the best of our knowledge, this is one of the first reports to sequence directly from animal samples using ONT and the first report that uses viral target enrichment using ONT with human and animal samples.

Detecting viruses and obtaining full-length genomes directly from samples has several benefits. Firstly, it enables the detection of viruses without *a priori* knowledge, thereby allowing the detection of novel and unexpected viruses. For example, we detected full- or near full-length genomes of bocavirus pig/SX/China/2010, porcine kobuvirus SH-W-CHN/2010/China and porcine respirovirus 1 [24,25] in this study. Secondly, it can be used to detect co-infections, such as norovirus GII.4 and the previously untypeable enterovirus A71 in sample H3. Thirdly, due to the untargeted nature of SMg and the 58% variation within the ViroCap panel [11], both approaches do not rely solely on primer-target identity. The latter can be problematic for highly diverse and evolving RNA viruses, such as PRRSV or enteroviruses [7]. Using NGS, we were able to characterize enteroviruses that were previously untypeable using conventional targeted Sanger sequencing in our human cohort. Detection and characterization are essential in revealing prolonged infections (particularly in vulnerable patients), following immunosuppressive drugs and provide crucial patient management information for infections with unknown etiologies or co-infections. Finally, full-length genomes can be used to predict antiviral resistance *in silico*—e.g., the susceptibility of the detected IAV H1N1 to neuraminidase inhibitors.

Genome coverage and sequencing depth (number of supporting reads) are essential to attain high-quality genome sequences. In this study, ViroCap increased the percentage of viral reads from 1.17% to 55.12% using ONT sequencing. ViroCap has been reported previously to improve viral detection in patient samples [11,12]. In this study, MV resulted in an overall better genome coverage in the human samples compared to NV (Table 3), despite a much lower sequencing depth (Table S1). Furthermore, ViroCap on the MinION had a considerably higher percentage of viral reads, compared to ViroCap on the NextSeq (Figure 2). This could be due to the Illumina workflow, in which cDNA is fragmented before generating libraries and performing hybridization. Therefore, more non-viral short fragments are likely to compete with viral cDNA for probe binding. Unfragmented long non-viral cDNA on the ONT is more likely to contain longer regions that are not binding (or have weaker interactions) to the probes. These whole fragments are then captured less often and washed away. Unfragmented long non-viral cDNA will therefore be competing less for probe binding, resulting in a more efficient viral enrichment.

Despite the improved viral sensitivity using ViroCap, a Ct 30 enterovirus was not detected on either platform from a fecal sample (sample H2). However, in sample H3 (another fecal sample), ONT with TSC covered 39% of the EV-A71 genome (Ct 29). Similarly, Illumina covered 40% of the EV-A71 genome in sample H3, suggesting a detection cut-off in fecal samples. Fecal samples are notoriously difficult to sequence in an untargeted fashion, due to the vast amount of nucleic acid background. Consequently, even a fecal sample with a low Ct of 21 (sample H4) resulted in only 35% genome coverage of CV-A22 with SMg. TSC was therefore required to obtain both a better sequencing depth and whole-genome coverage, enabling a more accurate viral classification.

Similarly to human samples, viral metagenomic sequencing has also been applied to farm animal samples. In a recent systematic review, only one publication used an ONT platform to sequence directly from animal samples, while Illumina was the predominant platform [26]. Pigs are the most frequently sequenced farm animals [26]. This is likely due to several global emerging swine viruses such as African Swine Fever [27], PRRSV [28] and zoonotic viruses such as IAV [29], which have been emerging in recent decades. Indeed, we detected an IAV that was closely related to a previously sequenced sample from a Dutch child presenting with a severe acute respiratory infection and requiring oxygenation [29]. Furthermore, sequencing of pig samples in this study revealed a highly diverse virome (Table 3). NV detected porcine bocavirus 5, which was not detectable in either M or MV, likely due to the former’s higher sequencing output (Table S1). Although M and MV detected the same number of viruses in the animal samples, MV yielded the highest number of full-length genomes, similarly to the human samples. NV had, on average, a higher overall genome coverage compared to MV in the detected viruses within the animal samples.

Sequencing errors remain a drawback in ONT platforms. Even in very high sequencing depths (≥1457), ONT achieved an average consensus accuracy of 99.44% (Table 4). This is likely the result of ONT sequencing errors which have not occurred randomly. As a result, high sequencing depths might not always improve the accuracy of consensus sequences.

The animal samples highlight an inherent limitation of TSC panels, as highly divergent or new viruses which are neither present in the panel nor share homology with viruses covered in the panel will not be enriched (Table 3). This is particularly relevant as the animal virome is less explored than the human virome. While these viruses were not enriched, they were still detectable, albeit with less genome coverage, such as porcine sapelovirus 1 (Table 3). ViroCap did enrich clinically significant viruses such as IAV and PRRSV substantially. Nevertheless, a simple shotgun approach proved to be very suitable for the virome characterization in the animal samples in this study.

Inherent limitations of TSC are the increased hours of sample handling and significant hybridization time before sequencing. Reported hybridization times in targeted sequence capture can range up to 72 h [12] or 16–20 h in the standard protocol. To reduce the turnaround time, we evaluated a hybridization time of 20 min. We found that a 20 min hybridization time resulted in a higher percentage of viral reads compared to 20 h. However, several viral species were underrepresented compared to the 20 h hybridization time, and, as a result, this hybridization time was not explored further. However, this study does show the potential of shorter incubation times, particularly if time is an essential factor. A further limitation of this study is the use of DNase treatment to improve viral sensitivity of RNA viruses in SMg. As a result, only actively transcribed DNA viruses would be expected to be detectable. Additionally, mapping with 80% nucleotide identity could result in the mapping of unknown or closely related viruses, particularly in highly divergent or complex samples [30]. With the established protocol for Illumina libraries, pooling multiple sequence libraries per capture reaction decreases costs and ensures an efficient workload, compared to running samples individually. In this study, we found that barcode primers in the ONT PCR barcoding kit were not specific enough for re-amplifying captured library pools. As a result, we observed significant crosstalk and an impossibility to demultiplex. Consequently, individual sample capture was used with 50% of the recommended ViroCap reagents to decrease the cost per reaction. Pooling of sequencing libraries prior to TSC using the ONT PCR barcoding kit might be feasible with the design of custom primers to enforce specificity during captured library pool re-amplification.

## 5. Conclusions

This study has shown that TSC on ONT platforms can be an alternative to Illumina sequencing, particularly if rapid results with few samples are required. Furthermore, ONT platforms can offer great flexibility as flow cells can be washed and re-used. ViroCap substantially improved the viral sensitivity and reduced background noise in the human samples considerably, increasing both genome coverage and sequencing depth compared to SMg. Although ViroCap did improve the sequencing depth for clinically relevant animal viruses such as influenza A virus, SMg alone was shown to have a higher overall genome coverage. Importantly, viruses that did not exist on the ViroCap panel were still detectable. Developing and evaluating new viral enrichment approaches applicable to both human and animal samples may prove crucial for the surveillance, detection and characterization of known and unknown viral infections.

## Figures and Tables

**Table 1:** Description of human and animal sample information.

Sample ID	Sample Type	qPCR Target	Ct Value	Symptoms	Sampling Date
H1	Flocked swab nasopharynx	Enterovirus	25	Fever, dyspnea and coughing	09/2018
H2	Fecal	Enterovirus	30	Chronic diarrhea	05/2016
H3	Fecal	Norovirus Enterovirus	17 29	Fever, vomiting and abdominal pain	11/2018
H4	Fecal	Enterovirus	21	Fever, diarrhea and abdominal pain	10/2016
A1	Blood plasma	PRSSV ^1^	-	None	12/2017
A2	Blood plasma	PRSSV ^1^	26 (pool)	None	10/2018
A3	Blood plasma	PRSSV ^1^	25 (pool)	Respiratory	10/2017
A4	Nasal swab	SIV ^2^	19	Respiratory (closed enteral system)	10/2018

^1^ PRRSV, porcine reproductive and respiratory syndrome virus; ^2^ SIV, swine influenza virus. Abbreviations: H, human; A, animal; Ct, cycle threshold.

**Table 2:** Sensitivity of detected viruses in pooled (20 min and 20 h hybridization) and individual targeted sequence capture (TSC) reactions. Sequencing reads were normalized against the total number of reads.

Type	Detected Viruses	20 min (Pool) %	20 h (Pool) %	20 h (Individual) ^1^ %
Human samples (*n* = 4)	Coxsackievirus A22	0.001	0.004	0.192
	Enterovirus A71	0.00004	0.00011	0.00028
	Enterovirus D68	0.003	0.027	0.775
	Norovirus GII.4	1.42	4.77	10.56
Animal samples (*n* = 4)	Astrovirus wild boar (*n* = 2)	0.002	0.010	0.001
	Bocavirus pig	0.001	0.007	0.003
	Influenza A virus *	55.31	51.15	9.39
	Mamastrovirus 2	0.0004	0.0017	0.0003
	Pasivirus A1	0.002	0.010	0.053
	PERV ^2^ (*n* = 4)	10.63	11.83	11.34
	Porcine astrovirus 4	0.001	0.008	0.001
	Porcine bocavirus H18	0.009	0.029	0.016
	Porcine enterovirus B	0.0002	0.0002	4.75 × 10^−5^
	Porcine kobuvirus	0.49	1.35	0.28
	Porcine respirovirus 1 ^3^	0.019	0.064	0.033
	Porcine sapelovirus 1	0.0001	0.0019	0.0003
	PRRSV ^4^ (*n* = 3)	9.75	6.82	15.75
	Rotavirus C *	0.00004	0.00005	0.00007
	Ungulate tetraparvovirus 3	10.02	0.44	5.24
	Total viral reads %	87.66	76.53	53.64

^1^ Sequencing reads from the individual TSC reactions were concatenated into one single file to provide a comparison to the pooled samples. ^2^ PERV, porcine endogenous retrovirus. ^3^ Porcine respirovirus 1 is also known as porcine parainfluenza virus 1. ^4^ PRRSV, porcine reproductive and respiratory syndrome virus; * segments were combined into one single genome.

**Table 3:** Genome coverage (%) and sequencing depth of trimmed sequencing reads against the appropriate viral reference sequence.

ID	GenBank	Reference Length	Reference	Genome Coverage (%)			Average Sequencing Depth		
				M	MV	NV	M	MV	NV
H1	MH341731.1	7345	Enterovirus D68	89	100	96	2	3342	2664
H2	**─**	**─**	No virus detected	**─**	**─**	**─**	**─**	**─**	**─**
H3	MK073885.1	7555	Norovirus GII.4	99	100	100	55	44,148	374,921
H3	LR027546.1	7410	Enterovirus A71	20	39	40	<1	1	4
H4	DQ995647.1	7401	Coxsackievirus A22	35	100	85	<1	898	152
**Average (Human Samples)**				**61**	**85**	**80**	**14**	**12097**	**94,435**
A1	HM159246	8774	PERV ^1^ C	83	71	64	28	7536	608
A1	NC_038546	5114	Porcine hokovirus HK7	18	41	74	1	977	6893
A1	NC_035180	5533	Ungulate tetraparvovirus 3	100	100	96	773	35518	380,898
A2	NC_038537	4786	Bocavirus pig/SX/China	43	59	82	5	143	425
A2	NC_038538	5267	Porcine bocavirus P18	39	56	61	5	130	479
A2	HM159246	8774	PERV ^1^ C	100	100	92	408	37,379	22,903
A2	NC_025402	15396	Porcine respirovirus 1	79	85	98	9	75	980
A2	GU067771.1	15098	PRRSV ^2^ (Amervac)	89	100	100	21	21,836	11,949
A3	NC_016896	6707	Astrovirus wild boar	10	17	32	60	1	144
A3	NC_030653	10908	Atypical porcine pestivirus 1	100	20	100	69	<1	36
A3	NC_018226	6916	Pasivirus A1	91	78	63	38	243	478
A3	HM159246	8774	PERV ^1^ C	33	81	49	2	1627	62
A3	KT344816.1	15095	PRRSV ^2^ (GER09-613)	99	100	96	85	17,451	8459
A4	NC_016896	6707	Astrovirus wild boar	98	81	76	20	12	128
A4	NC_027711	6327	Dromedary astrovirus	83	44	38	5	5	23
A4	KY250316-23	13200	Influenza A virus *	100	100	100	1512	24,292	197,905
A4	NC_034974	6347	Mamastrovirus 2	83	39	51	4	5	22
A4	NC_023675	6639	Porcine astrovirus 4	99	82	65	18	12	151
A4	NC_023636	6500	Porcine astrovirus 5	46	41	76	1	1	4
A4	NC_016647	5076	Porcine bocavirus 5	0	0	12	0	0	4
A4	HQ540591	9182	PERV ^1^ A	73	96	58	1	7	6
A4	KY214435	8043	Porcine enterovirus b	94	21	85	5	0.4	6
A4	NC_016769	8210	Porcine kobuvirus	99	100	99	152	1457	4056
A4	NC_003987	7491	Porcine sapelovirus 1	98	59	66	3	2	26
A4	GU067771.1	15098	PRRSV (Amervac) ^2^	20	93	100	<1	10	66
A4	MN102366-75	18286	Rotavirus A pig *	4	2	10	<1	<1	<1
A4	NC_003985	7117	Teschovirus A	23	41	62	<1	1	13
**Average (animal samples)**				**67**	**63**	**71**	**120**	**5508**	**23,582**

^1^ PERV, porcine endogenous retrovirus; ^2^ PRRSV, porcine reproductive and respiratory syndrome virus; * Influenza A virus and rotavirus A segments were concatenated into a single contig, respectively. Near full-length genomes (>94% coverage). Abbreviations: H, human; A, animal; M, MinION; MV, MinION with ViroCap; NV, NextSeq with ViroCap.

**Table 4:** Similarity of ONT consensus sequences compared to Illumina consensus sequences.

Detected Virus	Percentage Identity (%)	Average Sequencing Depth (MV)
Norovirus GII.4	99.89	44,148
Influenza A virus *	99.88	24,292
Enterovirus D68	99.83	3342
Ungulate tateraparvovirus 3	99.37	35,518
PRRSV ^1^	98.95	17,451
Porcine kobuvirus	98.71	1457
**Average**	**99.44**	**21,035**

^1^ PRRSV, porcine reproductive and respiratory syndrome virus. * Influenza A virus segments were concatenated into a single contig.

## Supplementary Materials

The following are available online at https://www.mdpi.com/1999-4915/12/12/1358/s1, Table S1: Description of human and animal sample information with corresponding Illumina and ONT approaches: MinION (M), MinION with ViroCap (MV) and NextSeq with ViroCap (NV).
