# Supplementary material for: Assessment of Viral Targeted Sequence Capture Using Nanopore Sequencing Directly from Clinical Samples
Source: Viruses. 2020 Nov 27;12(12):1358. doi: 10.3390/v12121358 (PMC7759923; doi:10.3390/v12121358)
Supplement: Supplementary file 1 [file viruses-12-01358-s001.pdf]

**Table S1.** Description of human and animal sample information with corresponding Illumina and ONT approaches: MinION (M), MinION with ViroCap (MV) and NextSeq with ViroCap (NV).

| Sample ID | Approach | Total reads (trimmed) | Average sequence length (bp) | Average quality score |
|---|---|---|---|---|
| H1 | M | 400,045 | 601 | 9.32 |
| | MV | 405,861 | 619 | 10.71 |
| | NV | 26,283,927 | 2x76 | 35.72 |
| H2 | M | 248,570 | 762 | 9.15 |
| | MV | 509,945 | 763 | 10.70 |
| | NV | 15,751,626 | 2x76 | 35.76 |
| H3 | M | 289,919 | 770 | 9.18 |
| | MV | 576,312 | 748 | 11.15 |
| | NV | 21,680,167 | 2x76 | 35.74 |
| H4 | M | 374,101 | 570 | 9.69 |
| | MV | 673,011 | 693 | 10.95 |
| | NV | 20,042,629 | 2x75 | 35.79 |
| A1 | M | 475,457 | 1.028 | 9.65 |
| | MV | 387,513 | 860 | 10.87 |
| | NV | 36,399,004 | 2x76 | 35.73 |
| A2 | M | 200,087 | 642 | 9.60 |
| | MV | 1,136,036 | 818 | 10.99 |
| | NV | 21,117,574 | 2x76 | 35.20 |
| A3 | M | 128,940 | 873 | 9.59 |
| | MV | 381,625 | 794 | 11.04 |
| | NV | 8,970,518 | 2x76 | 35.58 |
| A4 | M | 567,188 | 809 | 9.24 |
| | MV | 430,525 | 818 | 11.25 |
| | NV | 60,152,651 | 2x76 | 35.69 |
